# Preamble and Constitution of the Rock River Medical Society

**Published:** 1846-08

**Authors:** 


### PART IV.—EDITORIALS.

### ARTICLE XIV.

### PREAMBLE AND CONSTITUTION OF THE ROCK RIVER MEDICAL SOCIETY.

The undersigned, judging from the experience of the past, believing that association for the mutual improvement and protection of the professions of Medicine and Surgery, if properly conducted, may result in the same benefits to the associated, as to Agriculture and the Mechanical Arts.

And, whereas, these professions are not protected by any legal enactments, but left, as we believe they should always be, to protect themselves, and to manage tneir own affairs, being willing to trust our improvement to our own exertions: We, therefore, the Physicians, Surgeons, and Apothecaries of Northern Illinois and Southern Wisconsin, do resolve to associate ourselves together as a Medical Society, and for the rule, regulation and government of the same, have adopted the following

### CONSTITUTION.

Art. 1. This Association shall be called the Rock River Medical Society.

Art. 2. All Surgeons, Physicians, and Apothecaries, within the State of Illinois and Wisconsin Territory, may become members by signing their names to this Constitution, and paying the entrance fee hereafter named.

Art. 3. The objects of this Association shall be mutual improvement in the various branches of Medical, Surgical and Pharmaceutical knowledge.

Art. 4. The Officers of the Society shall be a President, 2 Vice Presidents, a Secretary, Treasurer, and three Censors, who shall hereafter be elected on the third Tuesday in May of each year, to perform such duties as are usually performed by like officers in similar societies, said officers to act till their successors are chosen.

Art. 5. The Society shall keep a common seal, and give to its members, (signed by the President, and countersigned by the Secretary,) sealed diplomas of membership.

Art. 6. Each member shall pay fifty cents each year on the third Tuesday in May.

Art. 7. The majority of members present at any annual meeting, may make and adopt such by-laws for its regulation and government, touching the qualifications of members, medical ethics, tariff of prices, &c. &c., as the majority may from time to time think necessary.

Art. 8. This Constitution may be altered, amended or added to by a majority of the members present, at any annual meeting.

Art. 9. There shall be an annual meeting of the society on the third Tuesday in May in each year, at 1 o'clock, p. m., at such place as the society may direct, and other meetings from time to time as may be found necessary and profitable.

Art. 10. There shall be delivered before the society at its annual meeting in each year, at least two essays on some medical subjects, by members of the society, fair copies of which shall be left at the disposal of the society by the authors.

Art. 11. Reports of interesting cases may be read at any meeting of the society, copies of which shall be left at the disposal of the society.

Art. 12. Any member of the society, after being heard in his own defence, may at the annual meeting be expelled for ungentlemanly or unprofessional conduct, by a vote of two-thirds of the members present.

### BY-LAWS.

Your committee, appointed to draft by-laws &c. for the regulation and government of the Rock-River Medical Society, respectfully beg leave to report the following:—

Art. 1. At each meeting of this society, after the members shall have been called to order by the officer whose duty it shall be to preside, the following order of proceedings shall be observed. 1st. The reading of the records of the last meeting, by the secretary. 2d. The admission of new members. 3d. Reading the treasurer's report. 4th. Reading the the president's address. 5th. Reading essays. 6th. Reports of cases, and interesting facts. 7th. Miscellaneous business. 8th. Election of officers.

Art. 2. No person shall be entitled to membership in this society, unless he produce evidence of being a graduate of some regular medical school—a licentiate of some properly constituted medical society, or pass a satisfactory examination before the board of censors of this society.

ART. 3. All debates and all other proceedings of this society shall be conducted according to parliamentary usages.

ART. 4. Eight members shall constitute a quorum, but a less number may organize to adjourn.

ART. 5. The officers of this society shall be elected by ballot, and a majority of votes shall determine the choice.

ART. 6. At every meeting of the society, the president shall appoint 2 members to read original essays on some medical subjects, at the next succeeding meeting.

ART. 7. It shall be the duty of the members to keep a record of any important cases that may occur in their practice, and all facts of peculiar interest connected with the profession of medicine, and report the same from time to time.

ART. 8. No address or paper read before the society shall be published without the consent of the author.

ART. 9. No member of this society shall be subject to a trial for misconduct, according to art. 12th of the constitution, until the secretary has given him, at least, three months notice.

ART. 10. Special meetings may be called by the President, with the concurrence of either of the Vice Presidents, whenever they may deem it necessary.

ART. 11. No money shall be drawn from the treasury without an order signed by the President and countersigned by the Secretary.

ART. 12. Any eminent medical man, residing without the limits of this society, may be elected an honorary member, by a vote of three fourths of the members present at any regular meeting.

A. M. CATLIN, M. D.,
*Chairman of the Committee.*

---

### MEDICAL ETHICS.

RULE 1. It is the duty of every medical practitioner to treat his patients with steadiness, tenderness and humanity, and to make due allowances for that mental weakness which usually accompanies bodily disease. Secrecy and delicacy should be strictly observed in all cases in which they may seem to be peculiarly required.

2. The strictest observance of temperance cannot be too strongly inculcated on the minds of the practitioners of medicine and surgery; a clear and vigorous intellect and a steady hand, being absolutely necessary to the successful practice of those branches of medical science.

3. Unfavorable prognostications should never be made in the presence of patients; yet, should there seem to be immediate danger, it becomes the duty of the medical attendant to apprise the patient's friends of that circumstance.

4. In every instance in which one physician has been called on to visit the patient of another, a consultation with the former medical attendant should be proposed. Consultation in difficult cases should always be recommended, and the physician called on for that purpose, should always pay the greatest degree of respect to the practitioner first employed, and allow him the privilege of delivering all the directions agreed upon.

5. Special consultations are sometimes wished for; in such cases, the physician called on, should carefully guard against paying another visit, unless he should be requested to continue his services by the patient, or some of his friends.

6. When one physician is called on to visit the patient of another in his absence, or during short indispositions, he should not manifest a wish to continue in attendance any longer than the physician first called on should be able to resume the charge of the case, unless a continuance of his services should be expressly wished for by the patient or his friends.

8. Theoretical discussions should not be too freely indulged in consultations, as they frequently give rise to much perplexity, without any improvement in practice.

9. The junior physician in attendance should always deliver his opinion first, the others according to seniority, and a majority should decide; but in the event of a tie, the physician first in attendance, should give the casting vote in regard to the future treatment, and to him should be intrusted the future management of the case, unless the patient or his relations should object to his being continued.

10. Although the possession of a diploma honorably acquired, furnishes presumptive evidence of professional ability and entitles its possessor to pre-eminence in the profession, yet, the want of it should not exclude practitioners of experience and sound judgment from the fellowship and respect of the regular graduate.

11. In consultations, punctuality in meeting at the same time should be strictly observed, but the physician who first arrives, should wait for a reasonable length of time for the arrival of others. A minute examination of the patient, however, should not take place until one or more of the medical attendants are present, except in cases of emergency. All subsequent visits, should if practicable, be made by mutual agreement, and no medical discussion should take place in the presence of the patient.

12. Attendance on members of the profession or their families should always be gratuitous, but should not be officiously obtruded. Should the circumstances of the medical practitioner indisposed, enable him to make a recompense for medical services rendered to himself, his wife or family, it is his duty to do so, especially if he reside at a distance.

13. When one medical practitioner is called on to visit a patient whose recovery has been despaired of by the physician first in attendance, and the disease should afterwards terminate fatally under his management, he should avoid insinuating to the friends of the deceased, that if he had been called on a day, or a few hours sooner, he could have effected a cure. Such a course of conduct is highly reprehensible, and empirical in the extreme. And, in the event of the patient's recovery, such a person should not assume all the credit, as the cure might have been partly effected by the medicines prescribed before he took charge of the case.

14. The use of nostrums and quack medicines, should be discouraged by the faculty, as degrading to the profession, injurious to the health, and often destructive of life. Should patients laboring under chronic complaints, obstinately determine to have recourse to them, a reasonable degree of indulgence should be allowed to their credulity by the physician; but it is his sacred duty to warn them of the fallacy of their expectations, and the danger of the experiment, and the necessity of strict attention to the effect produced by them, in order that their bad effects, if any, should be timely obviated.

15. No physician should, either by precept or example, contribute to the circulation of a secret nostrum, whether it be his own invention or exclusive property, or that of another. For, if it be of real value, its concealment is inconsistent with beneficence and professional liberty, and if mystery alone give it value and importance, such craft implies either disgraceful ignorance or fraudulent avarice.

16. In all cases where diversity of opinion and opposition of interest give rise to controversy or contention between two or more members of the profession, the decision should be referred to a sufficient number of physicians, as they are frequently the only persons in the community capable of properly estimating the merits of the dispute. But neither the subject litigated, nor the decision thereon, should be communicated to the public, as individual reputation might suffer, and the credit of the profession generally be injured.

17. A wealthy physician, or one retired from practice, should refuse to give gratuitous advice, unless the danger of the case (the absence of the practising physician) or the poverty of the patient should warrant him in so doing. In all cases where he may be preferred, he should recommend a consultation with some one engaged in active practice. This rule should be strictly observed, as a contrary course is gratuitously depriving active industry of its proper reward.

18. When a physician is called on suddenly to visit the patient of another, in consequence of some unexpected or alarming change in the symptoms, he should adopt a temporary plan of treatment suited to present circumstances. He is not warranted in interfering afterwards, unless requested to take charge of the case, when he should propose an immediate consultation with the physician previously employed.

19. Physicians should never neglect an opportunity of fortifying, and promoting the good resolutions of patients suffering under the bad effects of intemperate lives and vicious conduct; and, in order that their counsels and remonstrances may have due weight, it will readily be seen, that they should have full claim to the blameless life and high moral character, which we have stated to be a necessary pre-requisite to an honorable stand in the profession.

*Names of the Members of the Rock River Medical Society:*

| | | | | |
|---|---|---|---|---|
| George Haskell, M.D., | 17 | March, | 1846. | Rockford. |
| Samuel G. Armor, M.D., | " | " | " | " |
| Lucius Clark, M.D., | " | " | " | " |
| George Hulet, M.D., | " | " | " | Winnebago co. |
| Charles Mandeville, M.D., | " | " | " | " |

| | | |
|---|---|---|
| A. M. Catlin, M.D., | 17 March, 1846. | Rockford. |
| J. C. Goodhue, M.D., | " " " | " |
| Eli Hall, M.D., | " " " | Winnebago co. |
| Alden Thomas, | " " " | " |
| Nathan H. Palmer, | " " " | " |
| Wm. H. Ealer, (Apothecary) | " " " | Rockford. |
| C. Martin, M.D., | 18 " " | Freeport. |
| David Goodrich, | 17 May " | Rockford. |
| A. E. Ames, M.D., | 19 " " | Roscoe. |
| A. Clark, M.D., | " " " | Beloit, W.T. |
| O. Everett, M.D., | " " " | Dixon. |
| C. Van Brunt, | " " " | Rockton. |
| Dexter G. Clark, M.D., | " " " | Beloit. |
| Daniel Ransom, M.D., | " " " | Belvidere. |
| W. W. Welch, M.D., | " " " | Inlet, Lee co. |
| Daniel Brainard, M.D., | " " " | Chicago. |
| R. S. Molony, M.D., | " " " | Belvidere. |
| J. B. Nash, M.D., | " " " | Dixon. |
| A. W. Benton, M.D., | " " " | " |
| L. Humphrey, (Apothecary) | " " " | Beloit. |
| S. L. Clark, M.D., | " " " | |
| Edward Mead, M.D., | " " " | Geneva, Ill. |
| Hurd, M.D., | " " " | Byron. |

Pursuant to adjournment, the Rock River Medical Society met at the Court House, in Rockford, Winnebago Co. Ill., at 1 o'clock P. M. The President, J. C. Goodhue, M. D., in the Chair; Geo. Hulet, M. D., one of the Vice Presidents, being absent, Eli Hall, M. D., was appointed Vice President, pro tem.

J. C. Goodhue, M. D., President of the Society, then delivered an address on the passed history and present prospects of the profession, in the west.

The committee appointed to report a code of by-laws and medical ethics, then made their report, which was adopted.

On motion of Geo. Haskill, M. D., it was resolved, that each member furnish the secretary with a concise account of his vouchers, place of birth, &c. &c.

On motion of Prof. Mead, it was resolved, that a committee of four be named by the Chair, whose duty shall be to report to the Society at its next meeting, the present state of the insane population of Illinois: which committee was filled with the following named gentlemen; Prof. E. Mead, G. Haskill, M. D., A. Clark, M. D., and D. Martin, M. D.

After several questions of general interest to the profession, had been discussed at considerable length, without any special action on them, the Society proceeded to ballot for officers for the ensuing year; the following gentlemen received a majority of all the votes as follows, viz:

| | |
|---|---|
| For President, | J. C. GOODHUE, M. D. |
| For Vice Presidents, | R. S. MOLONY, M. D.<br>O. EVERETT, M. D. |
| Secretary & Treasurer, | S. G. ARMOR, M. D. |
| Censors, | G. HASKILL, M. D.<br>J. C. GOODHUE, M. D.<br>S. G. ARMOR, M. D. |

The Chair then appointed R. S. Molony, M. D. and O. Everett, M. D., to address the Society at its next meeting.

On motion of Prof. Brainard, it was resolved, that the proceedings of this meeting, the constitution, by-laws and medical ethics, be published in the Illinois & Indiana Medical and Surgical Journal.

On motion of S. G. Armor, M. D., it was voted to procure fifty copies of the above, for the use of the Society.

On motion of W. W. Welch, M. D., it was resolved that the thanks of the Society be and are hereby tendered to Dr. Goodhue, for his able and interesting address, and that he be requested to furnish a copy, or so much of it as relates to medical quackery and medical education, to the Illinois & Indiana Medical and Surgical Journal, for publication.

The President then requested Prof. Brainard, to deliver an address before the Society at 7 o'clock, P. M.: at which hour Prof. Brainard addressed the Society, on the subject of the improvements in surgery, with great credit to himself, and much to the gratification of the members of the Society.

The Society then adjourned, to meet at Dixon, Lee County, Ill., at one o'clock P. M., on the third Tuesday in October, next.

SAMUEL G. ARMOR,

*Secretary R. R. M. S.*

Rockford, Winnebago Co., Ill. May 19, 1846.

